# Continuous dynamics in behavior reveal interactions between perceptual warping in categorization and speech-in-noise perception

**DOI:** 10.3389/fnins.2023.1032369

**Published:** 2023-03-01

**Authors:** Gavin M. Bidelman, Jared A. Carter

**Affiliations:** ^1^Department of Speech, Language and Hearing Sciences, Indiana University, Bloomington, IN, United States; ^2^Program in Neuroscience, Indiana University, Bloomington, IN, United States; ^3^School of Communication Sciences and Disorders, University of Memphis, Memphis, TN, United States; ^4^Hearing Sciences – Scottish Section, Division of Clinical Neuroscience, School of Medicine, University of Nottingham, Glasgow, United Kingdom

**Keywords:** categorical perception, gradient perception, hysteresis, mouse-tracking, speech-in-noise perception

## Abstract

**Introduction:**

Spoken language comprehension requires listeners map continuous features of the speech signal to discrete category labels. Categories are however malleable to surrounding context and stimulus precedence; listeners’ percept can dynamically shift depending on the sequencing of adjacent stimuli resulting in a warping of the heard phonetic category. Here, we investigated whether such perceptual warping—which amplify categorical hearing—might alter speech processing in noise-degraded listening scenarios.

**Methods:**

We measured continuous dynamics in perception and category judgments of an acoustic-phonetic vowel gradient via mouse tracking. Tokens were presented in serial vs. random orders to induce more/less perceptual warping while listeners categorized continua in clean and noise conditions.

**Results:**

Listeners’ responses were faster and their mouse trajectories closer to the ultimate behavioral selection (marked visually on the screen) in serial vs. random order, suggesting increased perceptual attraction to category exemplars. Interestingly, order effects emerged earlier and persisted later in the trial time course when categorizing speech in noise.

**Discussion:**

These data describe interactions between perceptual warping in categorization and speech-in-noise perception: warping strengthens the behavioral attraction to relevant speech categories, making listeners more decisive (though not necessarily more accurate) in their decisions of both clean and noise-degraded speech.

## Introduction

An important characteristic of perceptual systems is that they form equivalence classes, assigning similar objects to the same membership despite variation in their physical properties (Goldstone and Hendrickson, 2010). Categorization is particularly salient in auditory processing and speech perception—although not all speech is perceived in a categorical manner (cf. Schouten et al., 2003). Forming categorical units allows listeners to downsample the auditory world and map continuous (and otherwise infinite) variations in the acoustic space into invariant linguistic-phonetic units necessary for speech-language processing. Indeed, categorical hearing plays a critical role in normal speech acquisition (Eimas et al., 1971; Vihman, 1996) and learning the grapheme-to-phoneme mapping essential for reading and writing skills (Werker and Tees, 1987; Mody et al., 1997). When identifying speech sounds along an acoustic-phonetic continuum, listeners show three hallmarks that denote categorical hearing: (1) an abrupt flip in category percept resulting in stair-stepped identification functions that inflect around a “category boundary,” (2) higher discrimination sensitivity to sounds between vs. within category; and (3) slower decisions speeds when labeling tokens near the boundary due to higher category ambiguity (Liberman et al., 1967; Pisoni, 1973; Harnad, 1987a; Pisoni and Luce, 1987).

An extreme view of categorization is that once established, internalized speech equivalence classes are invariant to context. In such schools of thought, surrounding sounds immediately preceding or following have no bearing on how listeners assign a token to a category (Liberman et al., 1957). These universalist views (Harnad, 1987b) suggest categorical boundaries are innate (e.g., Rosen and Howell, 1987) or occur naturally due to acoustic and/or neurophysiological discontinuities imposed by constraints in auditory processing (Harnad, 1987b). Under this innate-sensitivity hypothesis, sound representations in the brain’s auditory map might self-organize due to non-uniformities in cell firing between exemplar vs. non-exemplar sounds (cf. within vs. between category tokens) (Guenther and Gjaja, 1996). In this vein, cortical neurons show marked changes in their temporal discharge patterns across categorically perceived speech continua (Steinschneider et al., 1999, 2003).

On the other hand, there is ample evidence to suggest category representations are not strictly bottom-up manifestations of the acoustic space (e.g., Bidelman et al., 2013; Alho et al., 2016; Bidelman and Walker, 2017), but rather, are malleable to top-down influences. This is most noticeable in biasing effects, when individuals perceive a different category depending on the surrounding context^1^ or sequencing of stimuli, resulting in warping of the perceptual space and a location shift to their perceptual boundary (e.g., Pisoni, 1975; Diehl et al., 1978; Ganong, 1980; Francis and Ciocca, 2003; Wong and Diehl, 2003; Holt and Lotto, 2010; Cao et al., 2012; Bidelman et al., 2021). Such top-down effects can be described in terms of cognitive-representational systems which act on or modulate “low-level” sensory processing. However, an alternate view, at least for stimulus history effects on behavior, can be cast in the framework of nonlinear dynamical systems (Tuller, 2005). These systems exploit knowledge of their recent state history to modulate their response accordingly, with their output often in a constant state of flux.

A specific form of perceptual warping is most readily seen when the order (i.e., recent history) of stimuli on short time scales modulates how observers both see and hear current events (Tuller et al., 1994; Liaci et al., 2018; Sayal et al., 2020). Category warping is especially prominent at perceptual boundaries, where different patterns of behavioral identification can result for otherwise identical speech sounds (Tuller et al., 1994, 2008; Nguyen et al., 2009; Carter et al., 2022). For example, warping is evident when people classify an otherwise identical speech continuum presented in sequential (tokens delivered from one end to the other) vs. random order (Repp and Crowder, 1990; Tuller et al., 1994). Under sequential presentation, listeners often maintain their percept longer than expected, continuing to report the same category beyond their usual perceptual boundary. This results in a clear change in the inflection point of the sigmoidal identification function. For example, when categorizing sounds drawn randomly from an acoustic-phonetic continuum, there is usually a specific stimulus along the gradient where listeners show 50% identification. This marks the location where the heard category shifts from one percept to another and the so-called “category boundary.” When tokens are instead presented serially from one end of the continuum to the other (e.g., stepping from Tk1→Tk7 or Tk7→Tk1), the inflection point of identification shifts, suggesting a movement in where listeners perceive the category boundary. Leftward vs. rightward shifts can occur depending on whether listeners lag or anticipate their labeling reports relative to the direction of stimulus presentation. In the present study, we focus on the general phenomenon of perceptual warping, describing any context-dependent movement away from the nominal perceptual boundary (for directional effects, see Tuller et al., 1994; Carter et al., 2022). However, a specific lagging in percept beyond the expected categorical boundary is sometimes referred to as hysteresis. Such movement is presumably due to a bias favoring what has already been heard vs. what is to come (e.g., Macmillan et al., 1988). Indeed, under more ambiguous conditions that give rise to uncertainty, one possible strategy is for observers to maintain their previous response (Hock et al., 1993; Chambers and Pressnitzer, 2014). In speech perception, both stop consonant and vowel continua produce perceptual warping, though the warping is typically stronger for more ambiguous speech sounds like vowels (Studdert-Kennedy et al., 1970; Carter et al., 2022). In the framework of nonlinear dynamic systems, the warping of percepts toward a continuum endpoint can be described as an “attractor state” (Tuller et al., 1994). Collectively, these findings demonstrate phonetic speech categories flexibly update depending on the surrounding context of adjacent signals (Repp and Liberman, 1987).

Emerging evidence also suggests that forming categories might benefit speech perception in noisy listening conditions. Theoretically, once an equivalency between stimuli is formed, irrelevant variations among them can be deemphasized (Goldstone and Hendrickson, 2010). Based on this premise, we have hypothesized that hearing speech in a categorical mode (a more abstract level of coding) might help aid degraded speech perception since continuous features of the signal (e.g., within category cues, and physical features of the noise itself) can be largely discarded once category membership is established (see Bidelman et al., 2020). Supporting this notion, we have demonstrated speech categories are surprisingly robust to acoustic interference, diminishing only at very severe noise levels [i.e., negative signal-to-noise ratios (SNRs)] (Bidelman et al., 2019, 2020; Lewis and Bidelman, 2020). These behavioral results are bolstered by neuroimaging data which reveal the brain’s encoding of speech is not only enhanced for sounds carrying a clear phonetic identity compared to their phonetically ambiguous counterparts but that category members are actually more resistant to external noise (Bidelman et al., 2020). Larger resilience of category-level cues to noise is further supported by studies in both the auditory and visual domains (Gifford et al., 2014; Helie, 2017). Indeed, gradient (non-categorical) perception is not associated with speech-in-noise listening performance (Kapnoula et al., 2017), suggesting that while listeners do have simultaneous access to continuous, within-category cues (Pisoni and Lazarus, 1974; Pisoni and Tash, 1974; Spivey et al., 2005; Huette and McMurray, 2010), they do not readily exploit them when parsing speech in degraded conditions (cf. Kapnoula et al., 2017). Thus, both the construction of perceptual objects and natural discrete binning process of categorization might enable category members to “pop out” among a noisy feature space, thereby facilitating speech in noise processing (e.g., Nothdurft, 1991; Pérez-Gay Juárez et al., 2019; Bidelman et al., 2020). That is, having an established category might provide an attractor state, which acts as a landing point for perception. This notion is supported in spoken word recognition, where real words and high-frequency words are more successfully perceived in noise than pseudowords or low-frequency words (e.g., Rosenzweig and Postman, 1957; Pisoni, 1996).

One method of assessing listeners’ continuous dynamics in perceptual processing is with mouse-tracking (Spivey et al., 2005; Dale et al., 2007; Huette and McMurray, 2010). In these paradigms, listeners are presented tokens along a perceptual-continuum and are asked to categorize the stimulus trial by moving the mouse to either side of the screen which contains one of two category labels (e.g., “A” or “B”). The paradigm contrasts typical two alternative forced choice (2AFC) identification because it allows for the logging of a continuous motor response between the time of stimulus presentation to termination of the behavioral decision; such granularity is lost in the static nature of 2AFC tasks. For example, examining hand movement trajectories during a spoken language task, Spivey et al. (2005) showed that the shape of participants’ movements varied depending on whether words were of the same (e.g., “candle” and “candy”) vs. distinct (e.g., “candle” and “jacket”) phonological cohort. Cohort words had similar acoustic-phonetic properties that produced more “bowed” (i.e., less direct) paths that bifurcated the two response targets. This suggests listeners start with two concurrent lexical activations that eventually subside to their category decision as they accrue acoustic-information over time. Mousetracking has been similarly used to investigate perceptual dynamics and parallel processing in the categorization of pictures, words, and colors (Dale et al., 2007; Huette and McMurray, 2010).

In the present study, we assessed the intersection of two lines of inquiry into the perceptual organization of speech, examining how warping in auditory perception and category-level abstraction might enhance speech recognition in noise-taxing situations. We used mousetracking to measure continuous dynamics in listeners’ categorization of speech sounds. Tokens along an acoustic-phonetic vowel continuum were presented in random vs. serial order to invoke more/less perceptual warping. Additionally, we varied SNR to induce more/less listening difficulty. Warping of the perceptual space produced by warping enhances the strength of categorical speech percepts (Carter et al., 2022). Categories are also more resilient to noise than continuous features of the speech signal (Bidelman et al., 2019, 2020). Thus, we posited an interaction of SNR and token order, whereby the strengthening of categories via perceptual warping would enhance speech perception, particularly in noise (e.g., Kapnoula et al., 2017, p. 1595). Such findings would suggest that perceptual warping interacts with noise-degraded speech identification by strengthening the perceptual attraction to relevant phonetic categories.

## Materials and methods

### Participants

We recruited *N* = 30 young adults via digital crowdsourcing to participate in the online experiment. Remote testing was used due to COVID-19 and related institutional restrictions on in-person testing for research. One person’s data were lost due to technical error in logging, resulting in a final sample of *N* = 29 (12 male, 17 female; age: 25.2 ± 4.1 years). All reported normal hearing sensitivity by self-report. All but 4 participants were right-handed. Each had obtained at least a collegiate level of education (17.9 ± 2.0 years). All but one was a native speaker of American English; the other reported being a Persian-English bilingual fluent in English. Most had some formal musical training (4.7 ± 5.2 years). However, music training was not correlated with response measures (see section “Results”). Participants were paid for their time and gave informed consent in compliance with a protocol approved by the Institutional Review Board at the University of Memphis.

Listeners downloaded and ran the behavioral task (described below) on their personal computer. The paradigm was coded in MATLAB 2020a (The MathWorks, Inc; Natick, MA) and compiled into a standalone executable application for local runtime deployment. Limited information was also logged on each participant’s hardware configuration to ensure, to the degree possible, system uniformity. All participants ran 64-bit PC workstations [Windows 10 (x25); Windows 7 (x4)] from various manufacturers [Lenovo (7); Acer (1); HP (8); Dell (5); Microstar (4); Gigabyte-Tech. (4)] and reported compliance wearing headphones throughout the task (12 earbuds; 17 misc. circumaural). Information on mouse hardware (e.g., trackpad vs. standalone mouse) was not available and was therefore expected to contribute some amount of noise in our measurements.^2^

### Stimuli

*Speech continuum.* We used a synthetic 7-step vowel continuum spanning from /u/ to /a/ to assess perceptual warping and effects of noise on speech categorization (e.g., Bidelman et al., 2013). All stimuli were synthesized with a cascade formant synthesizer implemented in MATLAB similar to techniques described by Klatt and Klatt (1990). Vowels are advantageous here because their categorization is more ambiguous than other speech sounds (e.g., stop-consonants) (Pisoni, 1975), making them more prone to perceptual warping (Carter et al., 2022). Each token of the continuum was separated by equidistant steps acoustically based on varying only first formant frequency (F1). Tokens were 100 ms, including 10 ms of rise/fall time to reduce spectral splatter. Each contained identical voice fundamental (F0), second (F2), and third formant (F3) frequencies (F0: 100, F2: 1090, and F3: 2350 Hz). F1 was parameterized over 7 equal steps between 430 and 730 Hz such that the resultant stimulus set spanned a perceptual phonetic continuum from /u/ to /a/. Audio stimuli were sampled at 48,828 Hz, RMS amplitude normalized, and delivered binaurally through the user’s PC soundcard at 80% full-scale volume (set automatically via the program). Though not critical given the suprathreshold nature of our task, exact presentation level necessarily varied across listeners as sound calibration depended on the user’s specific PC audio configuration (e.g., soundcard, headphones). Estimated output level based on in-house laboratory calibrations was ∼70 dB SPL (through Sennheiser HD280 Pro headphones).

*Noise masking.* In addition to clean (no noise) conditions, this same speech continuum was presented in a noise block. Noise allowed us to assess whether perceptual warping is more/less prominent in challenging listening conditions. We set the SNR to 0 dB. This SNR balances listening effort during phoneme identification (Lewis and Bidelman, 2020) while still maintaining categorical hearing (Bidelman et al., 2019; Carter et al., 2022). For example, we have shown that more egregious SNRs (e.g., –5 dB)—where the noise level swamps the target speech—significantly reduces identification vowel identification performance and the brain’s differentiation of category structure (Bidelman et al., 2020). 0 dB SNR was therefore a compromise to ensure listeners could still categorize the speech sounds while still potentially revealing subtle warping effects in noise-degraded listening conditions. The noise masker was a speech-shaped noise based on the long-term power spectrum (LTPS) of the vowel set (Bidelman et al., 2020). LTPS noise was presented continuously so it was not time-locked to the phoneme presentation, providing a constant backdrop of acoustic interference during the noise block (e.g., Alain et al., 2012; Bidelman and Howell, 2016; Bidelman et al., 2018).

### Task procedure

There were six experimental conditions with fully crossed manipulations of noise (clean, 0 dB SNR noise) and token presentation order (random, forward, reverse). Each condition was presented in a different block. Block order was randomized within and between participants. For the random ordering, tokens were presented by random draw from the continuum. Sequential presentation involved delivering tokens ordered in either a forward (Tk1→Tk7) or reverse (Tk7→Tk1) direction along the continuum. Each sequence was repeated for the clean and noise blocks. There were 30 trials per token in each block (i.e., 210 trials per noise/order condition).

The identification task was otherwise modeled after similar mouse-tracking studies on perceptual categorization (Spivey et al., 2005; Huette and McMurray, 2010). On the start of each trial, listeners viewed a black screen on the computer monitor with the mouse cursor automatically (re)positioned at low center. An invisible horizontal threshold spanned the bottom of the window (1/8th the monitor’s vertical pixel resolution). Crossing the threshold initiated the trial: a target speech token was presented auditorily and visual representations of the endpoint tokens (i.e., “u” or “a”) were simultaneously displayed in each corner of the screen (see Figure 3). Participants were instructed to move the mouse vertically and continue movement toward the response area that best corresponded to the sound they heard. The trial ended—and the reaction time (RT) was logged—when the mouse hovered over an invisible box surrounding the user-selected vowel character. Following Huette and McMurray (2010), participants were instructed to “smoothly move to one response or the other” after initiating their movement and label the sound with a binary response (“u” or “a”) as quickly and accurately as possible. Mouse position was sampled every 10 ms (100 Hz). The identical task was repeated for each of the order/noise combinations. Breaks were offered between blocks to avoid fatigue.

### Data analysis

*Perceptual data.* Each individual’s identification scores were fit with a sigmoid function *P* = 1/[1+*e*^–^^β1^^(^*^x^* ^–^ *^β0^*^)^], where *P* is the proportion of trials identified as a given phoneme, *x* is the step number along the stimulus continuum, and β_0_ and β_1_ (the dependent measures) are the location and slope of the sigmoidal fit estimated using least-squares regression. Perceptual warping is indicated when the location of the perceptual boundary (β_0_) in phoneme identification shifts dependent on which serial direction speech tokens along the continuum are presented relative to randomly ordered presentation (Tuller et al., 1994; Carter et al., 2022). RTs were computed per token as listeners’ median response latency across trials. RTs outside 250–2,500 ms were deemed outliers (e.g., fast guesses, attentional lapses) and were excluded from analysis (Bidelman et al., 2013; Bidelman and Walker, 2017).

*Mouse-tracking data.* We first converted all mouse positions and screen measurements to normalized dimensions (i.e., converting x-y pixel coordinates to 0-1) to avoid potential differences in participants’ screen resolution. Left- and right-hand responses are mirror-images as either vowel endpoint (i.e., /u/ or /a/) can be treated as the target. Trials for which mouse trajectories were toward the left target (responses directed toward /u/ for Tk1 trials) were reflected across the vertical axis and averaged with those on the right (responses directed toward /a/ for Tk 7 trials) (Huette and McMurray, 2010). This effectively pooled endpoint tokens [i.e., mean(Tk1, Tk7)] allowing us to assess the curvature of response trajectories toward category prototypes and whether those patterns change with listening difficulty (noise) and stimulus sequencing (order). The degree of curvature was measured from each mouse trajectory as the area under the curve (a.u.c.), in pixels, computed between the actual trajectory and a straight line connecting its start and endpoint (Spivey et al., 2005; Dale et al., 2007; van der Wel et al., 2009). Curvature geometry was expected to increase for more categorically-ambiguous speech sounds such as those at the midpoint of the continuum (see Figure 3 in Viswanathan and Kelty-Stephen, 2018) or atypical exemplars (Dale et al., 2007).

Additionally, following Spivey et al. (2005), we measured the proximity of the mouse cursor to the category target over normalized time (averaged across left and right movements) via proportional Euclidean proximity [i.e., 1 – distance/max(distance)], computed using the *pdist2()* function in MATLAB. This measure provides a complementary way to quantify listeners’ continuous perceptual state and the degree to which their percept is attracted to the speech category over the time course of the trial. For details see Spivey et al. (2005). Condition effects between random vs. serial orders were tested across the entire time window (see Figure 4C) using a running (sample-by-sample) *t*-test (paired, *p* < 0.01). This approach is commonly applied in the EEG/ERP literature to assess differences in evoked potential waveforms (Guthrie and Buchwald, 1991) without the need for an *a priori* selection of analysis window.

### Statistics

We analyzed the dependent variables (i.e., psychometric function β_0_ and β_1_, RTs, mousetracking a.u.c.) using mixed-model ANOVAs in *R* (R Core Team, 2020) and the *lme4* package (Bates et al., 2015). Fixed effects were token (7 levels; Tk1-Tk7), SNR (2 levels; clean vs. 0 dB noise), and presentation order (2 levels; random, serial). Subjects served as a random effect. Multiple comparisons were corrected via Tukey–Kramer adjustments. Effect sizes are reported as $\eta_{p}^{2}$.

**FIGURE 1:**
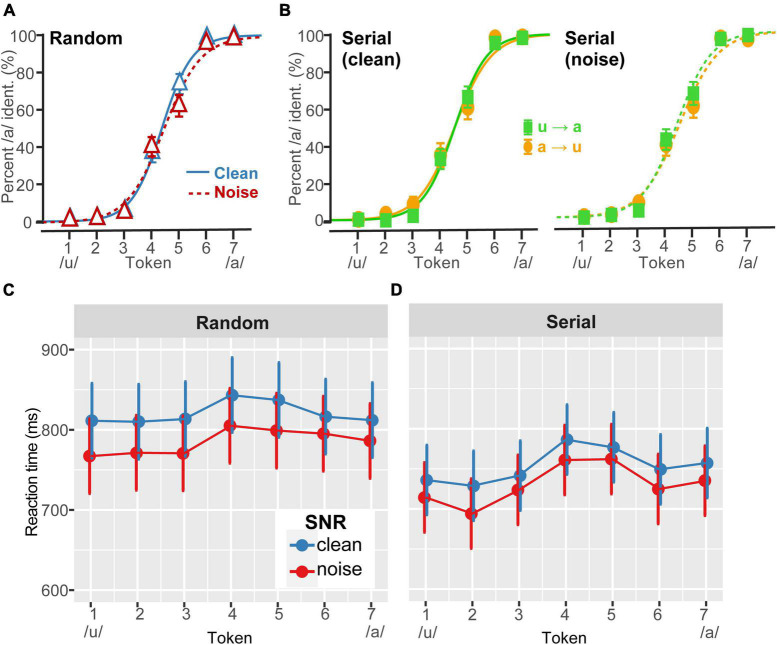
Perceptual warping in behavioral vowel identification. **(A)** Grand average perceptual psychometric functions for clean and noise-degraded speech identification. Noise (0 dB SNR) had minimal effect on categorical hearing. **(B)** Grand average comparison of (clean) vowel identification under forward (u→a) vs. reverse (a→u) serial ordering of the continuum (similar patterns were observed in noise). Warping was not prominent in the grand average data and is largely washed out at the group level (cf. the strong differences at the individual data, Figure 2). **(C,D)** Speech labeling speeds (RTs) for phenome identification. Listeners were faster at labeling continuum tokens under (i) noise vs. clean listening conditions and (ii) serial vs. random presentation. RTs also showed the typical slowing of responses near the continuum’s midpoint where category membership is ambiguous (Pisoni and Tash, 1974; Bidelman and Walker, 2017). Error bars = ±1 SEM **(A,B)**; 95% CI **(C,D)**.

**FIGURE 2:**
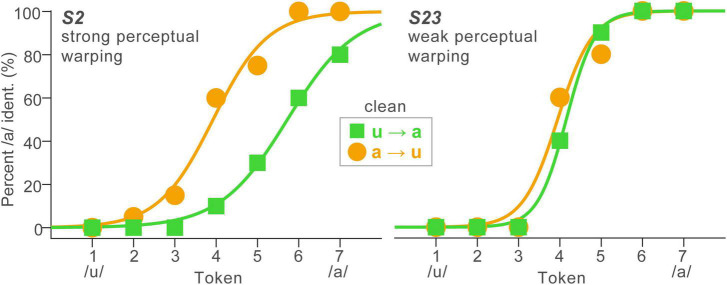
Perceptual warping in speech categorization is subject to stark individual differences. Identification functions for representative listeners (*n* = 2) who showed strong **(left)** and weak **(right)** perceptual warping. High influence listeners’ perceptual boundary shifts dramatically with stimulus order context, whereas low influence listeners show little movement in their category boundary with context.

We assessed relations between behavioral and mouse-tracking measures of perceptual warping via Pearson’s correlations between the mousetracking a.u.c. measures and behavior including (i) the magnitude change in perceptual boundary (β_0_) between random and serial orders (e.g., sigmoid shift in Figure 2) and (ii) RTs (Figures 1C,D) (cf. Carter et al., 2022). In these latter RT analyses, we only considered responses at Tk4 where perceptual categories are most ambiguous and susceptible to perceptual warping effects (e.g., Figure 2, present study; Carter et al., 2022). Separate analyses were run for the clean vs. noise conditions.

**FIGURE 3:**
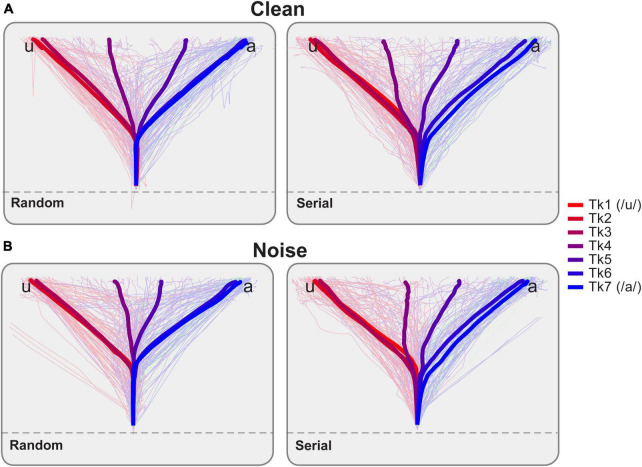
Mouse trajectories reflect continuous dynamics in vowel identification as a function of noise and stimulus ordering. Light traces = single trials, Bold thick traces = grand average tracks per vowel token. Forward and reverse directions are pooled for the serial order. Once participants moved the mouse across an invisible threshold on the screen (dotted line), a speech token was played. They then identified which vowel they perceived by moving toward either the “u” or “a” character presented on either side at the top of the display. Response tracks for **(A)** clean and **(B)** noise-degraded speech. Listeners’ response tracks were more direct toward endpoint tokens (i.e., those heard with a strong category identity; Tk1, Tk7). Midpoint tokens, which are more category ambiguous, elicited tracks which appear more centered on the screen, indicating listeners split their decisions between categories. Note also differences in the bowing of mouse trajectories for serial vs. random presentation order.

**FIGURE 4:**
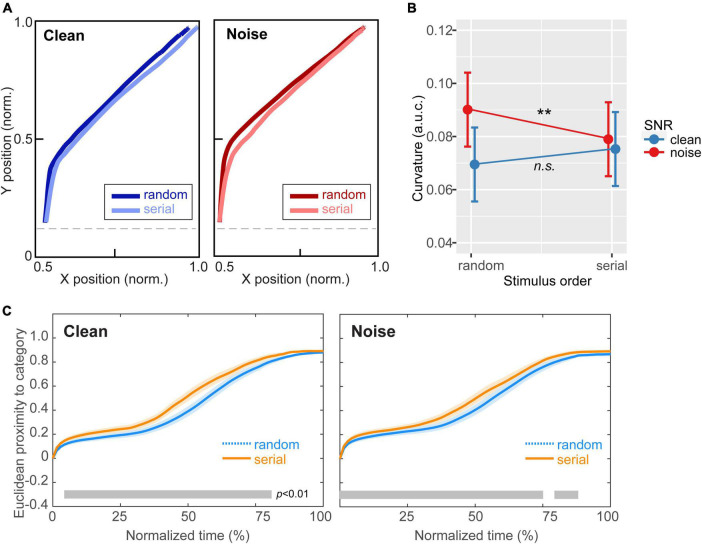
Continuous dynamics in speech categorization reveal perceptual warping helps hone categories in noise. **(A)** Mousetracks toward the category endpoint [mean of Tk1 (mirrored) and Tk7] for clean and noise-degraded speech for random and serial token ordering. **(B)** Track curvature was invariant to stimulus order for clean speech. However, amidst noise, mouse responses were less bowed (i.e., more directed) to the speech category under serial presentation. **(C)** Proximity of the mouse cursor to target speech over normalized time (Spivey et al., 2005). For both clean and noisy speech identification, the mouse remained closer to the ultimate selection in serial vs. random order, indicating increased perceptual attraction to the phonetic category (i.e., perceptual warping). However, stimulus order effects emerged earlier and persisted later in the time course of perceptual decision in the noise condition [gray bars = running t-test, *p* < 0.01, paired samples; (Guthrie and Buchwald, 1991)]. **p < 0.01. Shading = ± 1 SEM. Error bars = 95% CI.

## Results

### Behavioral identification

Psychometric identification curves are shown as a function of noise level and presentation order in Figure 1. As per our design, moderate noise weakened but did not overly hinder speech identification performance (Figure 1A). Visual inspection of the curves did not reveal strong effects of serial direction on the perceptual boundaries at the *group level* (Figure 1B), consistent with prior studies (Carter et al., 2022). This occurs because some listeners show different directions of perceptual warping, with some flipping their response before and some after the nominal categorical boundary depending on their individual listening strategy (for discussion, see Carter et al., 2022). Consequently, it might be argued that analyzing the psychometric data at the group level data is somewhat misleading. Hence, we favored individual level data in our subsequent analysis.

In stark contrast, shifts in the category boundary varied substantially across listeners, reminiscent of other individual differences including context (e.g., Ganong effect: Ganong, 1980; Myers and Blumstein, 2008; Lam et al., 2017; Bidelman et al., 2021) and perceptual warping effects observed during speech categorization (Carter et al., 2022). Despite these individual differences, response patterns were highly stable within listeners; a split-half analysis showed β_0_ boundary locations were strongly correlated between the first and last half of the task trials across orders and SNRs (*r* = 0.79, *p* < 0.0001). This suggests that while perceptual nonlinearities (i.e., β_0_ shifts) varied across listeners, response patterns were highly repeatable within individuals. At the individual level, some listeners showed strong perceptual state memory (hysteresis) while others showed little to no change in their category boundary with stimulus ordering (Figure 2). For example, *S2* showed strong warping, i.e., a preponderance of “a” responses for more tokens of the continuum when they were presented in the reverse direction (a→u); in contrast, *S23* showed very little displacement in category boundary (weak warping). Consequently, we pooled responses to forward and reverse directions for data reduction purposes in subsequent analysis. These qualitative observations were confirmed by an ANOVA, which showed psychometric slopes were invariant to SNR and stimulus order effects [*ps* > 0.25]. Perceptual boundary locations were also impervious to SNR and order [*ps* > 0.26].

In contrast, RTs were highly sensitive to all three stimulus manipulations. Decision speeds varied with noise [*F_1,1162_* = 53.40, *p* < 0.0001; $\eta_{p}^{2}$ 0.04], presentation order [*F*_1,1162_ = 226.82, *p* < 0.0001; $\eta_{p}^{2}$ 0.16], and token [*F*_6,1162_ = 11.54, *p* < 0.0001; $\eta_{p}^{2}$ 0.06], with no interactions (Figures 1C, D). The token effect was attributed to a slowing of RT speeds near the midpoint of the continuum where category membership becomes perceptually ambiguous (Pisoni and Tash, 1974; Bidelman and Walker, 2017). This inverted V-shape pattern in the RT data was observed at both SNRs for serial (contrast Tk4 vs. mean of others; clean: *p* < 0.0001; noise: *p* = 0.0002) but not random (clean: *p* = 0.045; noise: *p* = 0.076) presentation ordering. Overall, serial presentation also yielded faster RTs than random presentation (*p* < 0.0001), confirming a facilitation of decision speeds dependent on stimulus context. Responses were also faster in noise compared to clean speech (*p* < 0.0001). Taken together, the overall stronger categorical pattern and faster overall RTs for serial vs. random ordering corroborates the notion that serial presentation order facilitates speech categorization decisions (e.g., Carter et al., 2022).

### Mousetracking data

Raw mousetrack responses are shown as a function of vowel token, SNR, and presentation order in Figure 3. In general, listeners’ mouse tracks were more direct toward endpoint tokens (i.e., those heard with a strong category identity; Tk1, Tk7). For midpoint tokens (∼Tk 4), which are more category ambiguous, tracks were more sporadic and often bifurcated between response alternatives. That is, listeners split their responses 50% of the time resulting in paths that were more centered on the screen. Note also differences in the bowing of mouse trajectories from start to response termination for serial vs. random presentation order and for noise vs. clean speech.

Figure 4A shows mousetracks toward the category endpoint for clean and noise-degraded speech and random vs. serial token ordering. Visual inspection suggests that response trajectories were similar between stimulus orders under clean speech. However, for noise-degraded speech, listeners appeared to respond with more direct movement toward the category. An ANOVA conducted on mouse trajectory curvature (measured via a.u.c.) confirmed these observations; movements strongly varied with both SNR and stimulus presentation order [SNR x order: *F*_1,200_ = 8.90, *p* = 0.0032; $\eta_{p}^{2}$ 0.04]. Tukey-adjusted contrasts revealed this interaction was due to a differential order effect between noise conditions. Whereas curvature was invariant to order for clean speech (*p* = 0.15), noise-degraded speech elicited mousetracks that were less bowed for serial vs. random order (*p* = 0.0061) (Figure 4B). This suggests serial ordering produced response trajectories that were more strongly directed to the end category.

Figure 4C provides a complementary view of these data, illustrating time-varying Euclidian proximity of mouse movements from the ultimate category judgment as a function of normalized time (Spivey et al., 2005). This proximity measure describes listeners’ continuous perceptual state and the degree to which their percept is attracted to the speech category over the trial’s time course. For both clean and noisy speech identification, the mouse remained closer to the behavioral selection in serial vs. random order, indicating increased perceptual attraction to the phonetic category (i.e., perceptual warping). However, these stimulus order effects emerged earlier and persisted later in the time course of decision when categorizing speech in noise. The differential pattern indicates warping was (i) more prominent in acoustically challenging listening scenarios and (ii) strengthened the perceptual attraction to the relevant speech category.

**FIGURE 5:**
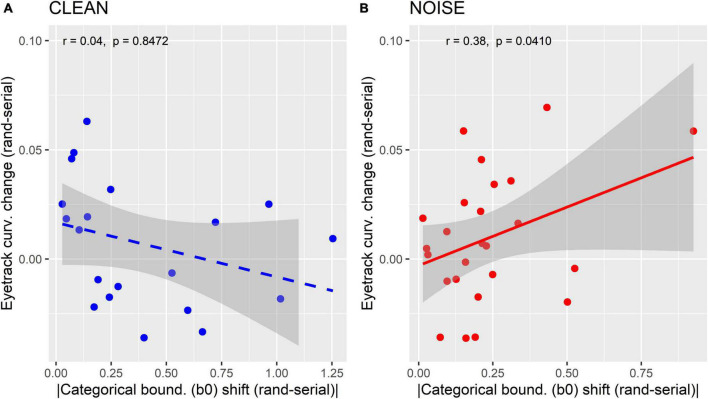
Correlations between behavioral and mousetracking measures of perceptual warping. **(A)** Clean condition. **(B)** Noise condition. Listeners with larger magnitude shifts in their psychometric boundary (β0) between random and serial token orders (indictive of stronger perceptual warping) show more salient change in the trajectory of their mousetracks. Mouse trajectories show straighter trajectories to the ultimate behavioral response under serial order. This brain-behavior relation is only observed for noise-degraded speech. Solid line = significant correlation; dashed line = n.s.; Shaded regions = 95% CI.

Figure 5 shows correlations between mouse-tracking and behavioral β_0_ measures separately for clean and noise-degraded speech. For these measures, we focused on the change in response from random to serial presentation to quantify the degree to which listeners’ mouse curvatures were related to their perceptual warping (e.g., change in perceptual boundary location). We found those with larger magnitude shifts in their psychometric boundary (β_0_) between random and serial token orders (indictive of stronger perceptual warping) showed more salient change in the trajectory of their mousetracks. Recall that mouse trajectories were more direct to the ultimate behavioral response under serial order (Figure 4B). This indicates that the degree to which listeners altered their response paths toward the ultimate behavioral category is predicted by stronger perceptual warping. These correlations were observed for noise (*r* = 0.38, *p* = 0.04) but not clean (*r* = 0.04, *p* = 0.85) speech. This further supports the notion that perceptual warping is more prominent in acoustically demanding scenarios. Correlations between mousetracking data and RTs were not significant (clean: *p* = 0.38; noise: *p* = 0.08).

Musical training might improve speech-in-noise processing (Parbery-Clark et al., 2009; Alain et al., 2014; Mankel and Bidelman, 2018; Bidelman and Yoo, 2020) and phoneme categorization (Bidelman et al., 2014; Bidelman and Walker, 2019). However, correlations between these behavioral measures and listeners’ years of musical training were not significant (all *p*s > 0.215). However, we note that our sample had an average of only ∼5 years of musical training, whereas studies reporting musician advantages in these processes typically include individuals with decades of training (Alain et al., 2014).

## Discussion

We measured listeners’ categorization of speech sounds along an acoustic-phonetic gradient where continua varied in (i) trial-to-trial presentation order (context) and (ii) signal clarity via changes in noise level. Mousetracking traced continuous dynamics in listeners’ behavioral responses as they first heard and then subsequently made categorical judgments on vowel identity. Our findings provide evidence that binning sounds into their category membership and changes in those category representations arising from perceptual warping facilitates noise-degraded speech identification (e.g., Bidelman et al., 2020).

Our data corroborate other moustracking studies that suggest information processing during categorization tasks reflects a probabilistic activation of multiple response outcomes (Spivey et al., 2005; Huette and McMurray, 2010; Viswanathan and Kelty-Stephen, 2018). This is directly observed in the continuous dynamics in listeners’ mouse movements; responses began largely bifurcated between outcomes but were quickly directed toward the end hearing (decision) over the trial. More directed response paths were observed for tokens heard with strong (endpoint) vs. weak (midpoint) category. These data show that short-term stimulus history warps corresponding phoneme judgments. Reminiscent of “perceptual magnet” accounts of CP (Iverson and Kuhl, 2000) and “attractor states” in nonlinear dynamics systems (Tuller et al., 1994), we found predictable (serial) stimulus presentation facilitated identification, yoking perception toward category endpoints compared to unpredictable (random) presentation. Behavioral speeds were also faster when labeling endpoint (strong category) vs. midpoint (weak category) tokens (i.e., RT_Tk1/7_ < RT_Tk4_). This is presumably due to the *decreased* listening effort involved in processing sounds that are easily assigned to a phonetic category vs. those which are phonetically ambiguous and bifurcate in percept (Lewis and Bidelman, 2020). Such slowing near the categorical boundary can be described in terms of more ambiguity in the decision process (Viswanathan and Kelty-Stephen, 2018). More interestingly, our RT data showed an overall stronger inverted V-shape pattern and overall faster response speeds for serial vs. random ordering. These findings extend studies showing that perceptual warping induced by serial presentation facilitates speech categorization decisions (Carter et al., 2022) by showing similar effects for noise-degraded speech.

Our mouse tracking data also support this notion. Listeners appeared to initially move the mouse upward and then diverged to the left/right when they had enough information to do so. Indeed, the longest initial (vertical) segment was in the noise/random condition, which is presumably due to limited *a priori* information about the token’s identity for randomized ordering, with the noise adding additional uncertainty. Conversely, the shortest vertical track was observed in the serial-clean condition. Here, the serial order may have provided early information about the token’s likely identity. Consequently, the differential “bowing” effects observed in the mouse trajectory data might reflect the degree of stimulus information that is available to guide response movements. The interaction of the two factors indicates that that predictability is less useful for clean vs. noisy stimuli. Under this interpretation, the observed perceptual warping might be somewhat epiphenomenal, a mere function of the uncertainty a listener faces in any given trial. Serial order reduces uncertainty which would tend to counter the increased uncertainty inflicted by noise. Indeed, under more ambiguous conditions that give rise to uncertainty, one possible strategy is for observers to maintain their previous response, resulting in a form of perceptual warping known as hysteresis (Hock et al., 1993; Chambers and Pressnitzer, 2014).

Our results also corroborate notions that category-level cues provide easier readout to brain processing (Pisoni and Tash, 1974; Guenther et al., 2004; Bidelman et al., 2013, 2020; Reetzke et al., 2018). However, we extend prior studies by demonstrating categorical percepts might also be more impervious to surface-level degradations that can corrupt speech recognition (Gifford et al., 2014; Helie, 2017; Bidelman et al., 2019, 2020). Comparisons between categorization under clean vs. noise-degraded listening conditions revealed listeners easily labeled speech even at unfavorable SNRs, confirming the mere process of binning sounds in categories helps fortify the speech signal against noise interference (cf. Bidelman et al., 2020).

Short-term sequential effects in speech perception are known to modify category boundaries (Diehl, 1975; Diehl et al., 1978, 1985; Eimas and Miller, 1978; Healy and Repp, 1982). The data here show these movements in the perceptual boundary were subject to stark individual differences (e.g., Figure 2). Compared with behavior when stimuli are presented in random order, serial presentation seems to lead some listeners to expect an imminent change of category, while others seem to expect the phonetic category to remain the same from trial to trial. This is consistent with recent reports on rapid vowel categorization demonstrating there is stark individual variation in the degree to which listeners experience perceptual warping (Carter and Bidelman, 2023). The mechanisms behind such differential patterns are not well understood and such variability is generally masked at the group level. However, while perceptual warping varies between people, response patterns are highly repeatable within a listener (present study; Carter et al., 2022). The faster RTs for serial over random presentation is consistent with prior studies on perceptual warping and hysteresis (Carter et al., 2022), and suggests a quasi-priming effect whereby responses to adjacent tokens are facilitated by the preceding (phonetically similar) stimulus.

Perceptual warping in categorization could be realized via phonetic “feature detectors” (Eimas and Corbit, 1973) that occupy and are differentially sensitive to different segments of the acoustic-phonetic space. Tunable detectors would tend to create quasi “acoustic foveae” that naturally build categories via overrepresentation of the stimulus space near protypes (Rozsypal et al., 1985). Adaptation studies—in which continuum sounds are presented repetitively and or in serial order as done here (Eimas and Corbit, 1973; Miller, 1975)—suggest movement of the category boundary is explained by one detector becoming more desensitized from fatigue, thereby causing a boundary shift in the direction toward the un-adapted detector at the polar end of the continuum (Rozsypal et al., 1985). As confirmed empirically, larger boundary shifts would be expected for less strongly categorized continua (Rozsypal et al., 1985), e.g., vowels vs. stop consonants (Altmann et al., 2014; Carter et al., 2022), acoustically degraded speech (Bidelman et al., 2019, 2020), and for ambiguous speech tokens as shown here and previously (Ganong, 1980; Gow et al., 2008; Myers and Blumstein, 2008; Lam et al., 2017; Noe and Fischer-Baum, 2020; Carter et al., 2022). Alternatively, displacements in the psychometric function’s inflection point could occur if stimulus context moves the category boundary toward the most likely perceptual candidate.

Whether or not the observed warping effects are pre-perceptual (due to sensory-perceptual dynamics and warping) vs. post-perceptual (due to response and decisional biases) remains undetermined given the purely behavioral nature of our data. There is no clear temporal division between “sensory encoding” and “decision/post-perceptual” stages of speech processing. Still, several pieces of evidence suggest that early, sensory processes might drive the observed warping effects. First, we found mouse tracks diverged almost immediately (<100 ms) after stimulus presentation (Figure 4C), much earlier than listeners’ collective RTs (∼800 ms). This is well within the timeframe (<250 ms) with which speech categories begin to emerge in auditory-sensory brain activity (Bidelman et al., 2013; Mahmud et al., 2021). Contextual effects due to stimulus history have also been observed in both animal (Lopez Espejo et al., 2019) and human (Carter et al., 2022) superior temporal gyrus. Ongoing work from our group has further demonstrated that speech representations in auditory brainstem, as indexed by frequency-following responses (FFRs), show perceptual warping effects like those observed cortically (Carter and Bidelman, 2023). Collectively, such findings argue that warping effects for speech begin early and likely at a pre-perceptual stage of processing.

Surprisingly, identification was faster in noise compared to clean speech. On the contrary, we would have anticipated slower speeds in more challenging listening conditions (cf. Price and Bidelman, 2021). The direction of this effect is unclear, but it is possible participants might have guessed more in noise leading to more rapid RTs at the expense of less accurate labeling (e.g., Yellamsetty and Bidelman, 2018). However, this explanation seems unlikely since identification percentages did not change appreciable with noise. Alternatively, faster RTs for noisy speech might reflect increased arousal, which could speed up RTs. Still, we note the SNR-RT effect was small in size so these accounts remain speculative.

Of particular interest is the finding that perceptual warping effects were more prominent under noise relative to clean speech. Mouse trajectory curvature was more susceptible to stimulus order effects and response paths remained closer to the ultimate category judgement and developed faster under serial vs. random presentation. Both the more direct and faster motor responses indicate an increased perceptual attraction to the category supplied by context-dependent perceptual warping that is more prominent in noise. These findings suggest warping improves the internal speech code by strengthening the perceptual attraction to the most relevant acoustic-phonetic category (Carter et al., 2022) and reducing decision ambiguity (Viswanathan and Kelty-Stephen, 2018). As such, we describe a new interaction between perceptual warping in speech perception and benefits to speech-in-noise listening.

The degree to which listeners show categorical vs. gradient perception might reflect the strength of phonological processing, which could have ramifications for understanding both theoretical accounts of speech perception and certain clinical disorders that impair sound-to-meaning mapping (e.g., dyslexia; Werker and Tees, 1987; Joanisse et al., 2000; Calcus et al., 2016). On one hand, graded/continuous perception might be advantageous for speech perception in noise since it would allow listeners to access all acoustic information in the signal, potentially allowing them to “hedge” their bets on what they are hearing in the face of ambiguity (Kapnoula et al., 2017). On the contrary, if a large portion of the perceptual space is corrupted by noise, hearing in discrete units might be preferrable to allow category members to “pop out” among the noise and facilitate speech processing (Nothdurft, 1991; Pérez-Gay Juárez et al., 2019; Bidelman et al., 2020). In support of the latter, empirical studies suggest that more gradient categorizers do not show better speech-in-noise performance (Kapnoula et al., 2017). Instead, category-level cues are robust to noise (Bidelman et al., 2019, 2020) and stimulus manipulations that amplify categorical hearing can enhance speech in noise processing (present study; Bidelman et al., 2019). Taken together, these findings suggest that while listeners have simultaneous access to continuous, within-category cues (Pisoni and Lazarus, 1974; Pisoni and Tash, 1974; Spivey et al., 2005; Huette and McMurray, 2010), they do not readily exploit them but instead rely on discrete speech representations to parse speech. Still, future studies are needed to test whether listening performance in realistic speech perception scenarios (e.g., “cocktail party” paradigms with spatially diverse, multi-talker soundscapes) (e.g., see pardigm in Bidelman and Yoo, 2020) is related to how well category information can be extracted (or suppressed) from concurrent speech streams.

The present work establishes a new link between perceptual nonlinearities and speech-in-noise processing. It has been suggested that deficits in speech categorization among certain developmental disorders might be more prominent in noise (Calcus et al., 2016). Consequently, we have speculated that assessing speech categorization under acoustically taxing demands might offer a more sensitive marker of impairment (Bidelman et al., 2020). Both perceptual warping and speech-in-noise aspects of hearing show considerable *inter-*subject (but less *intra*-subject) variability (present study; Song et al., 2011; Billings et al., 2013; Bidelman et al., 2018; Bidelman and Momtaz, 2021; Carter et al., 2022). Thus, it is tempting to infer that figure-ground deficits observed in some auditory and language-based learning disorders (Cunningham et al., 2001; Warrier et al., 2004; Putter-Katz et al., 2008; Lagacé et al., 2010; Dole et al., 2012, 2014) might result from a failure to flexibly warp category representations of the speech code. Future studies in clinical populations are needed to test this possibility.

## Data availability statement

The raw data supporting the conclusions of this article will be made available by the authors, without undue reservation.

## Ethics statement

The studies involving human participants were reviewed and approved by University of Memphis IRB, Protocol #2370. The patients/participants provided their written informed consent to participate in this study.

## Author contributions

GB designed the experiment and analyzed the data. JC collected the data. Both authors contributed to interpreting the results and writing the manuscript.

## Footnotes

1 By context we refer specifically to how local stimulus history (precedence) of time affects perceptual responses to otherwise identical speech tokens. In other types of speech perception tasks, “context” might also include spatial, linguistic, and/or other “top-down” signals (in addition to temporal events) that can affect behavioral responses (Viswanathan and Kelty-Stephen, 2018).

2 We can loosely infer mouse vs. trackpad use based on whether the model of the computer system in use was a desktop or laptop system. *N* = 19 used laptops, *N* = 6 used desktops, and *N* = 4 used an unknown system. Thus, the majority of participants ran the experiment on laptops, and consequently, likely used a track pad. However, this inference does not account for participants that may have used an external (e.g., USB) mouse with their device so these data should be regarded strictly as estimates.
